# Depletion of the Protein Hydration Shell with Increasing Temperature Observed by Small-Angle X‑ray Scattering and Molecular Simulations

**DOI:** 10.1021/jacs.5c13497

**Published:** 2025-12-11

**Authors:** Johanna-Barbara Linse, Hyun Sun Cho, Friedrich Schotte, Philip A. Anfinrud, Jochen S. Hub

**Affiliations:** † Theoretical Physics and Center for Biophysics, 9379 Saarland University, Saarbrücken 66123, Germany; ‡ Laboratory of Chemical Physics, National Institute of Diabetes and Digestive and Kidney Diseases, 2511 National Institutes of Health, Bethesda, Maryland 20892, United States

## Abstract

The hydration shell is an integral part of proteins since it plays key roles in conformational transitions, molecular recognition, and enzymatic activity. While the dynamics of the hydration shell have been described by spectroscopic techniques, the structure of the hydration shell remains less understood due to the lack of hydration shell-sensitive structural probes with high spatial resolution. We combined temperature-ramp small-angle X-ray scattering (*T*-ramp SAXS) from 255 to 335 K with molecular simulations to demonstrate that the hydration shells of the IgG-binding domain of Protein G (GB3) and the villin headpiece are remarkably temperature-sensitive. For proteins in the folded state, *T*-ramp SAXS data and explicit-solvent SAXS predictions consistently demonstrate decays of protein contrasts and radii of gyration with increasing temperature, which are shown to reflect predominantly temperature-sensitive, depleting hydration shells. The depletion is caused not merely by enhanced disorder within the hydration shells but also by partial displacements of surface-coordinated water molecules. Together, *T*-ramp SAXS and explicit-solvent SAXS calculations provide a novel structural view of the protein hydration shell, which underlies temperature-dependent processes such as cold denaturation, thermophoresis, or biomolecular phase separation.

## Graphical abstract

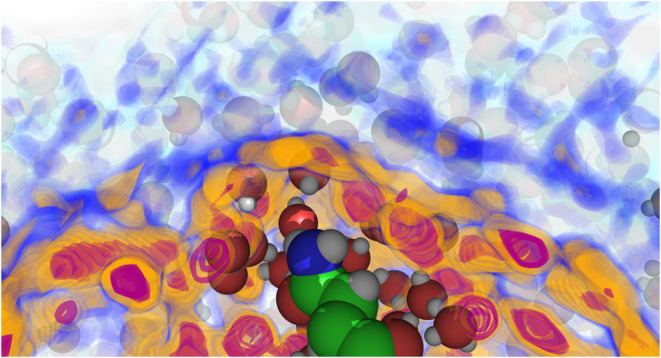

## Introduction

The hydration shell is an integral part of biomolecules since it mediates a wide range of biological functions. Water in the hydration shell of enzymes actively participates in hydrolytic enzymatic reactions, acid–base reactions, or proton transfer via the Grotthus mechanism.^1–3^ The hydration shell also orchestrates protein folding, ligand binding, or protein–protein recognition because such processes involve extensive rearrangements of protein–water and water–water interaction networks. Hydration-shell water exhibits different properties compared to bulk water. Techniques such as NMR, terahertz, sum-frequency generation, or inelastic neutron scattering spectroscopy have shown that the vibrational, rotational, and translational dynamics of hydration shell water are slowed down approximately two- to fivefold relative to those of bulk water.^4–18^

While the dynamics of the hydration shell have been extensively studied, its structural properties remain less well understood. X-ray and neutron crystallography provide insight into highly localized water molecules that coordinate with biomolecular surface residues;^19,20^ however, these techniques are largely blind to more dynamic water or to water in the second and third hydration layers. Small-angle scattering with X-rays or neutrons (SAXS/SANS) is sensitive to the hydration shell, but it yields data with low information content and low spatial resolution. Accordingly, SAXS/SANS has shown that many proteins exhibit hydration shells with an overall increased density relative to the bulk, which manifests in an increased radius of gyration (*R*_g_) relative to the *R*_g_ of the bare protein.^21,22^ Molecular dynamics (MD) simulations with explicit water corroborated these findings and explained the modified *R*_g_ values by an increase in hydration shell density of ∼6%.^23–26^ However, the influence of protein geometry, surface composition, pH, and temperature on the hydration shell remains poorly characterized, largely due to the paucity of experimental methods capable of probing the hydration-shell structure at atomic resolution.

Solvation of biomolecules is governed by a delicate balance of significant enthalpic and entropic contributions, which often mostly compensate to yield moderate solvation free energies. Because entropic effects are amplified at high temperatures, solvation is inherently temperature-dependent, with implications for biomolecules and soft-matter systems. For instance, the hydrophobic effect, which drives protein folding and micelle and lipid membrane assembly, is temperature-dependent and entropy-driven at 22 °C yet enthalpy-driven at 113 °C.^27–29^ Temperature-dependent solvation drives the unfolding of proteins at low temperatures (cold denaturation)^30,31^ as well as the collapse of intrinsically disordered proteins^32^ or liquid–liquid phase separation at high temperatures.^33^ Such effects would be at odds with the naive expectation that high temperatures would generally favor polymer disorder, thus highlighting the importance of hydration shell effects. The drift of molecules or beads along temperature gradients, an effect known as thermophoresis, thermodiffusion, or the Soret effect, is driven by the temperature dependence of the solvation entropy.^34,35^ The mechanisms by which such effects contribute to the response of biological systems to changing temperatures, for instance, during thermosensing by membrane channels or thermotaxis by bacteria, are not known. Additionally, the size and shape of detergent or polymer micelles are temperature-sensitive, which has likewise been associated with temperature-dependent solvation.^36,37^ Thus, structural insight into temperature-dependent solvation is essential to rationalizing a plethora of biological or soft-matter phenomena.

We combine temperature-ramp (*T*-ramp) SAXS experiments and MD simulations to reveal how temperature controls the hydration shell structure of two proteins in their folded state, the third IgG-binding domain of Protein G (GB3) and chicken villin headpiece (also termed HP35), which have been used extensively as model proteins for biophysical studies. We developed infrastructure on the BioCARS beamline at the Advanced Photon Source for acquiring SAXS data over a broad temperature range, spanning 257–393 K, which covers supercooled conditions up to unfolded proteins. To rationalize the temperature effects on our SAXS data by atomic means, we use all-atom MD simulations combined with explicit-solvent SAXS calculations.^25,38^ Our strategy is supported by recent findings that the overall hydration shell density obtained by MD simulations with many protein and water force fields aligns accurately with consensus SAXS/SANS data.^39,40^ Our *T*-ramp SAXS data and MD simulations are quantitatively consistent and demonstrate that, in the folded state, the protein hydration shell is remarkably temperature-sensitive, as shown by partial displacements of surface-coordinated water molecules.

## Results

Figure 1A (upper row) presents the three-dimensional electron density of the solvent around the GB3 domain, computed from an MD simulation with the TIP4P/2005 water model, which has been parametrized to reproduce water properties across a broad temperature range.^41^ By using position restraints on all heavy atoms, conformational fluctuations of solvent-exposed protein atoms were excluded, thereby yielding a spatially well-defined hydration shell with a first and a second hydration layer (orange and blue densities, respectively). The density maps reveal that increasing the temperature from 250 to 350 K leads to a partial loss of the hydration shell structure, as evidenced by the decreased densities of the first and second hydration layers. These simulations provide the atomistic rationale for interpreting the temperature-dependent scattering intensities observed in SAXS experiments.

**Figure 1:**
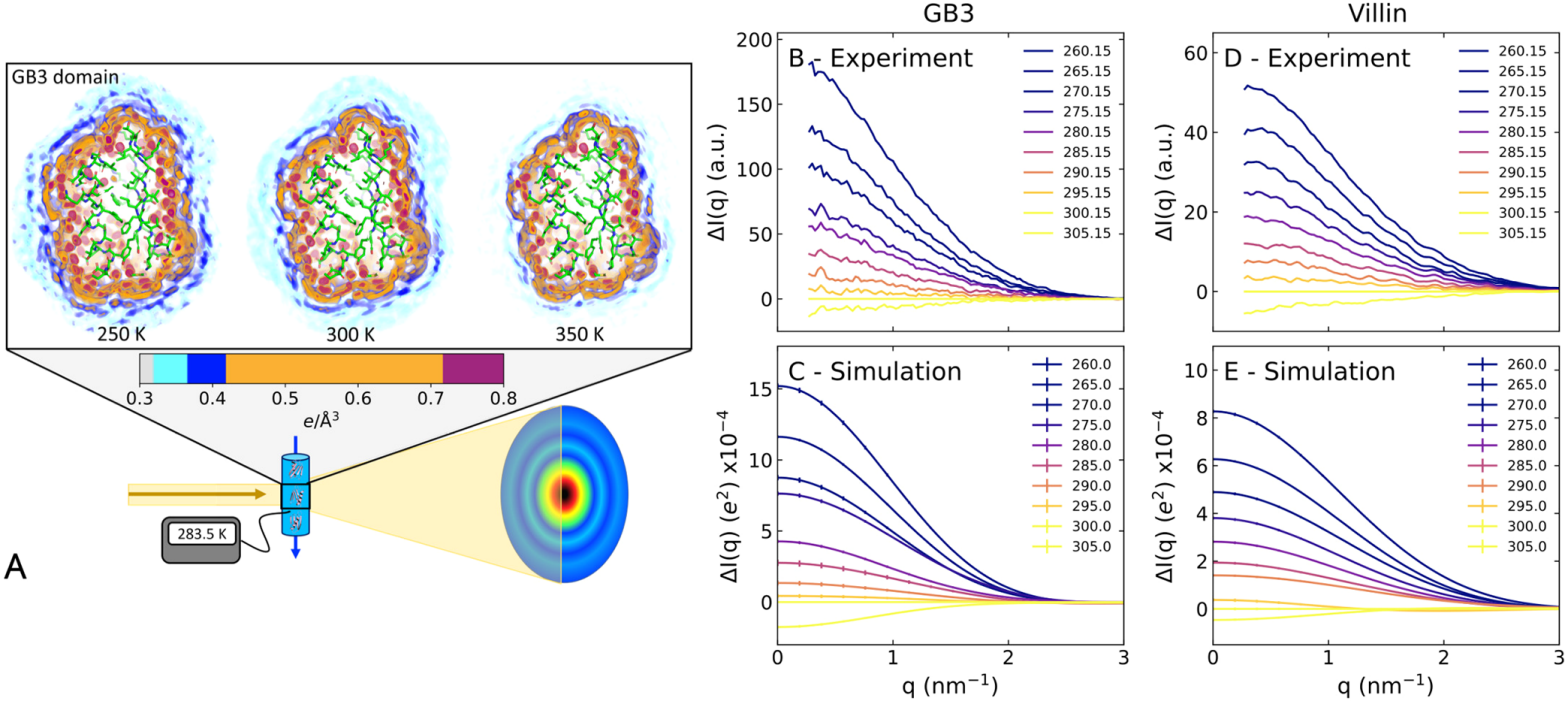
(A) Illustration of temperature-ramp SAXS experiments. Upper row: three-dimensional electron density around the GB3 domain at 250, 300, and 350 K from MD simulations with restrained heavy atoms. The protein is shown as sticks, and the solvent electron density is represented in colors ranging from cyan to purple, as indicated by the color bar, revealing the depletion of the hydration shell structure with increasing temperature. Scattering intensity difference relative to 300 K, Δ*I*(*q*), of the GB3 domain at temperatures spanning ∼260 to ∼305 K (see the legend) (B) from experiments or (C) from simulations. Δ*I*(*q*) for the villin headpiece (D) from experiments or (E) from simulations. Absolute SAXS curves are shown in Figures S1 and S2.

SAXS data for the GB3 domain and villin headpiece were acquired on the BioCARS beamline at the Advanced Photon Source using a *T*-ramp protocol that generates scattering images while repeatedly ramping the sample temperature between 257 K and up to 393 K. The SAXS curves of both proteins exhibited clear temperature dependence (Figures S1A and S2A). Thanks in part to the small volume of protein solution in the HF-etched, temperature-controlled capillary, the solution could be repeatably supercooled to 257 K without freezing. The temperature dependence of the SAXS curves is highlighted by the difference intensities Δ*I*(*q*) relative to the experimental reference temperature of 300.15 K (Figure 1B,D). In this study, we focus on two key parameters that are frequently obtained from the small-angle regime: (i) the forward scattering intensity, *I*_0_ = *I*(*q* = 0), which is given by the square of the contrast between the solute and the solvent (in number of electrons), scaled in the experiment by a concentration-dependent factor; and (ii) the radius of gyration, *R*_g_. These parameters were obtained via Guinier analysis: ln­(*I*(*q*)/*I*_0_) ≈ *q* ^2^ *R*_g_^2^/3, where *I*(*q*) is the SAXS curve and *q* is the momentum transfer. Specifically, Guinier analysis was carried out after extrapolating a series of concentration-dependent intensities to the infinite dilution limit, thereby removing modest effects from the structure factor at low *q* (Figure S3).

Our SAXS experiments revealed that the forward scattering *I*_0_ decreased by more than 20% for both the GB3 domain and the villin headpiece across the measured temperature range, demonstrating a decreasing electron density contrast that plateaued at ∼320 K for the GB3 domain and at ∼335 K for the villin headpiece (Figure 2A,D, black dots). The *R*_g_ values decreased by 0.4 Å for the GB3 domain and by 0.3 Å for the villin headpiece between 257.15 and ∼300 K (Figure 3A,C, black dots). This trend is followed by a slight increase in *R*_g_ up to ∼330 K, likely indicating enhanced conformational fluctuations, followed by sharp increases in *R*_g_, indicating protein unfolding. The temperatures of the sharp *R*_g_ increase are compatible with previously reported melting temperatures of the GB3 domain and villin headpiece.^42–44^

**Figure 2:**
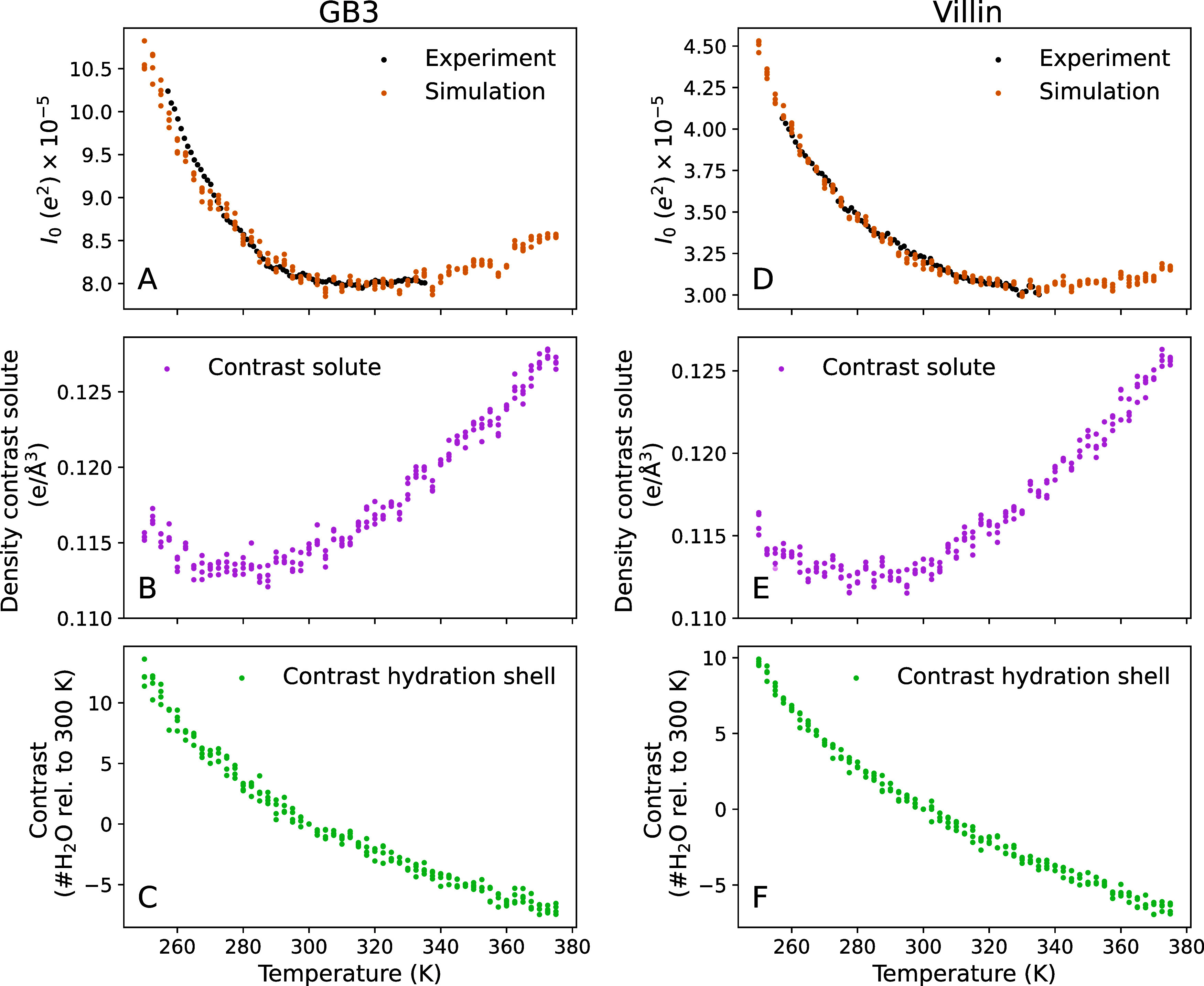
Forward scattering *I*_0_ and decomposition of the solute–solvent contrast into contributions from the bare protein and the hydration shell for the (A–C) GB3 domain and (D–F) villin headpiece. (A, D) *I*_0_ versus temperature from experiments (black) and backbone-restrained MD simulations with explicit-solvent SAXS calculations (orange). The experimental curves were scaled by a constant factor of the simulation data in the temperature range below 303 K. (B, E) Density contrast of the backbone-restrained bare protein from MD simulations, reflecting the temperature dependence of the solvent density. (C, F) Contrast of the hydration shell in the number of water molecules relative to 300 K. In all panels, colored dots indicate simulation results from four independent simulations per temperature. The analysis of panels (A–F) visualized as contrasts in the number of electrons is shown in Figure S5.

**Figure 3:**
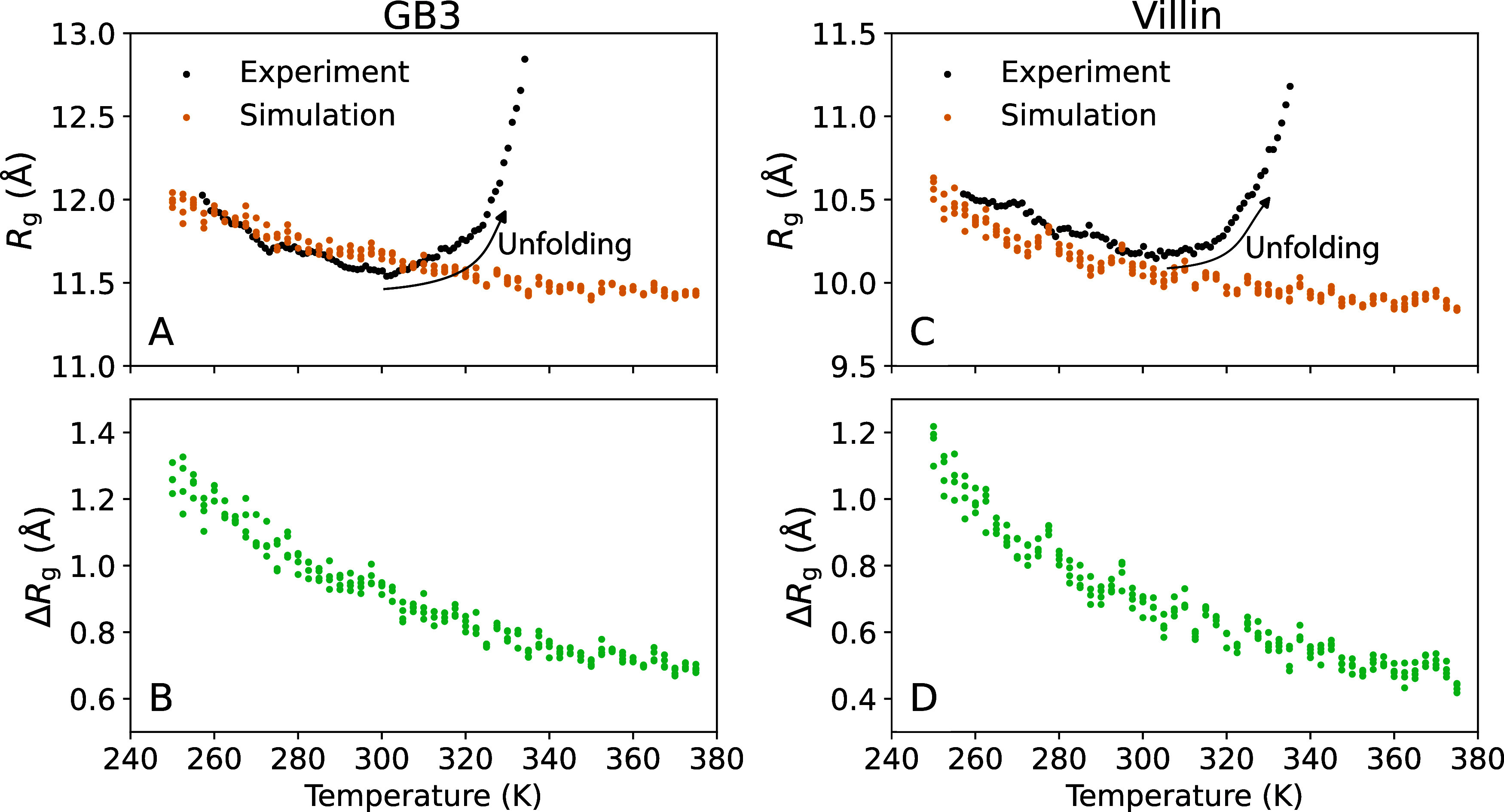
Radius of gyration *R*_g_ versus temperature for the (A, B) GB3 domain and (C, D) villin headpiece. (A, C) *R*_g_ from Guinier analysis from experiments (black) or backbone-restrained MD simulations (orange). (B, D) From MD simulations and explicit-solvent SAXS calculations, the difference Δ*R*_g_ between the *R*_g_ from Guinier analysis (including hydration shell contributions) and the *R*_g_ of the bare protein. A decrease in *R*_g_ with temperature in backbone-restrained simulations demonstrates the depletion of the hydration shell. Colored dots indicate simulation results from four independent simulations per temperature.

We hypothesized that the decreases in *I*_0_ and *R*_g_ between 257 and 300 K, where the proteins remain folded, reflect gradual depletions of the protein hydration shells. To test this, we carried out MD simulations of the GB3 domain and villin headpiece (Figure S4) over a temperature range of 250–375 K in steps of 2.5 K. SAXS curves were computed from MD simulations according to the WAXSiS method, thereby taking the explicit solvent into account.^25,45^ To isolate the effect of the solvent and to prevent unfolding, the simulations were carried out with position restraints on backbone atoms; thus, variations in the computed SAXS curve were purely caused by temperature-dependent variations in the hydration shell and excluded solvent densities.

In line with our SAXS experiments, the computed SAXS curves were temperature-dependent, as indicated by the absolute scattering curves (Figures S1B and S2B) and by the difference curves relative to 300 K (Figure 1C,E). The *I*_0_ and *R*_g_ values taken from the calculated curves via Guinier analysis are in good agreement with the experimental values in the ≤300 K regime, where the proteins remain fully folded (Figures 2A,D and 3A,C, yellow dots). The agreement is remarkable considering that, in our explicit-solvent SAXS calculations, neither the hydration shell nor the excluded solvent density was fitted to the experimental data. The agreement implies that (i) thermal expansion of the protein, which is excluded in our MD simulations with backbone restraints, has only a small effect on *I*_0_ and *R*_g_ in this temperature range; and (ii) the MD simulations with the TIP4P/2005 water model provide a realistic representation of the hydration shell structure across a broad temperature range.

To isolate the effect of temperature on the hydration shell, we decomposed the total contrast Δ*N*_e_ (in the number of electrons) into contributions from the protein and the hydration shell. The forward scattering follows *I*_0_ = Δ*N*_e_^2^, and the total contrast is

$$\Delta N_{e}=\Delta N_{e}^{\mathrm{prot}}+\Delta N_{e}^{\mathrm{hs}}$$

where Δ*N*_e_^hs^ is the contrast imposed by the hydration shell and Δ*N*_e_^prot^ = *N*_e_^prot^ – ρ_solv_ *V* ^prot^ is the contrast of the bare protein with the temperature-dependent solvent density ρ_solv_, *V* ^prot^ is the protein volume, and *N*_e_^prot^ is the number of electrons in the protein. Figure 2B,E shows the electron density contrast of the bare proteins, Δ*N*_e_^prot^/*V* ^prot^, reflecting purely the temperature dependence of the water density as the protein volumes are nearly fixed. Here, the convex shapes of the density contrast curves reflect the well-known maximum of the water density at 4 °C, with the density decreasing at both lower and higher temperatures. Critically, the contrast between the hydration shell and bulk water Δ*N*_e_^hs^ greatly decreases with increasing temperature, demonstrating a depleting hydration shell structure (Figure 2C,F). Specifically, relative to 300 K, the hydration shell of both the GB3 domain and the villin headpiece contains approximately ten additional water molecules at 250 K and five fewer water molecules at 360 K (Figure 2C,F). Over the entire temperature range simulated here, the hydration shells of the GB3 domain and villin headpiece lose 19 and 17 water molecules, respectively. We note that the quantitative decomposition of the Δ*N*_e_ into contributions from protein and hydration shell depends on the definition of the protein volume; thus, a different definition may lead to a constant shift between Δ*N*_e_^prot^ and Δ*N*_e_^hs^,^26^ which would, however, not alter the trends as a function of temperature. Together, this analysis supports our hypothesis that the experimentally and computationally observed decay of *I*_0_ reflects temperature-sensitive depletion of the hydration shell.

As a second indicator of the hydration shell, we computed from the simulations the increase of the radius of gyration due to the hydration shell, Δ*R*_g_ = *R*_g_ – *R*_g_^prot^, defined as the *R*_g_ from Guinier analysis relative to the *R*_g_^prot^ of the bare protein (Figure 3B,D). Recently, we found excellent agreement for Δ*R*_g_ values at room temperature obtained from consensus SAXS/SANS data and MD simulations using various protein force fields and water models, including the TIP4P/2005 water model employed here.^39,40^ According to the simulations, Δ*R*_g_ decays with increasing temperature by ∼0.6 Å, reflecting the diminishing hydration shell in line with the decreasing *I*_0_ discussed above. Considering that (i) *R*_g_^prot^ was fixed in simulations due to backbone restraints and (ii) *R*_g_ values from simulations agreed with experimental values in the *T* ≤ 300 K regime where the proteins remain folded (Figure 3A,C), this analysis suggests that the *R*_g_ decays in experiments up to 300 K are likewise signatures of depleting hydration shells with increasing temperature.

The results described above were obtained from simulations with restrained backbone atoms, thereby avoiding conformational transitions and isolating the effect of the hydration shell on the contrast and *R*_g_. To exclude the possibility that backbone fluctuations might influence our key findings, we carried out an additional series of simulations without restraints for the GB3 domain (Figure S9, red triangles). Whereas the results reveal slightly increased variability among different temperatures, as expected from enhanced protein fluctuations, GB3 remained folded within the simulation time, even at high temperatures, and the trends are conserved across the entire temperature range.

Having established that the temperature-dependent hydration shell contrast agrees between simulations and experiments, we used the simulations to gain structural and energetic insights into the hydration shell and its interaction with the protein. Visual inspection of the hydration shell densities confirms that the hydration shells are gradually depleted as the temperature increases (Movies S1 and S2 and Figure 1A). To resolve contributions from individual surface-bound water molecules, we computed the density differences relative to 300 K from an additional series of simulations with restraints on all heavy atoms, thereby suppressing side-chain fluctuations and leading to a spatially well-resolved hydration shell structure (Figure 4 and Movies S3 and S4). In addition to the overall depletion of the first and second hydration layers, these density difference maps reveal the loss of numerous localized solvent densities. Visual inspection showed that most of these localized density differences originate from molecules that are coordinated via hydrogen bonds with the protein, whereas a few density differences arise from water molecules trapped in small hydrophobic pockets (Figure 4, red and blue spots). Critically, localized densities from surface-bound water at low temperatures are not merely smeared out at higher temperatures but are partially lost. This finding is supported by the hydration shell densities as a function of distance from the van der Waals surface of the proteins, which reveal a partial loss of the first and second hydration shell density peaks (Figures S6A and S7A). These structural changes are accompanied by a ∼13% decrease in both protein–water interaction energies and the number of hydrogen bonds over the simulated temperature range (Figures S6B,C and S7B,C). Nevertheless, even at 350 K, the overall hydration shell structure with its pronounced first and shallow second peaks remains intact (Figures S6A and S7A), suggesting that the decrease of *I*_0_ and *R*_g_ reflects a partial but not complete loss of the protein–water coordination with increasing temperature.

**Figure 4:**
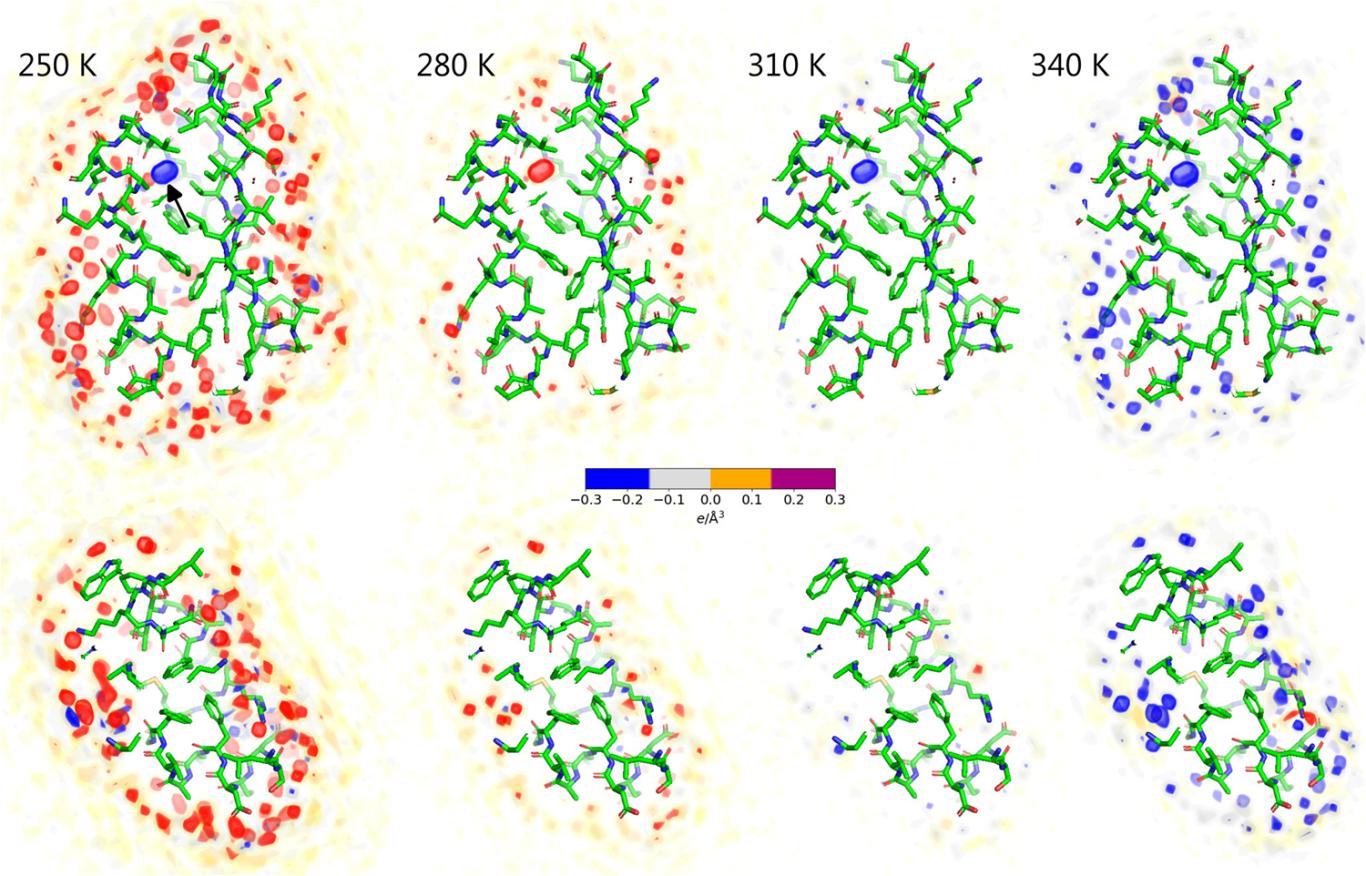
Electron density difference of the solvent relative to 300 K for the GB3 domain (upper row) and villin headpiece (lower row) for temperatures of (from left to right) 250, 280, 310, or 340 K. Densities were computed from MD simulations with restraints on all heavy protein atoms. Increased and decreased densities relative to 300 K are represented by red and blue densities, respectively (see the color bar), revealing the depletion of surface-bound densities with increasing temperature. The localized density (black arrow) refers to a randomly placed water molecule within a GB3 cavity, which is not exchanged with the bulk within the simulation time. Proteins are shown as sticks.

In addition to analyzing the protein–water coordination discussed above, we investigated how temperature affects the internal structure of the hydration shell. To this end, we computed the number of water–water hydrogen bonds within a distance of 9 Å from the protein surfaces of the GB3 domain and villin headpiece (Figures S6D and S7D), revealing a ∼20% decrease over the simulated temperature range. This trend, together with an increasingly smeared-out water–water radial distribution function within the hydration shell (Figure S8), demonstrates a considerable loss of internal water structure within the hydration shell, consistent with previous computational and spectroscopic studies.^46–49^ At high temperatures, the decrease in the number of hydrogen bonds is more pronounced in the hydration shell compared to the bulk solvent, suggesting that the hydration shell is more temperature-sensitive than bulk water (Figures S6D and S7D). Together, these analyses suggest that increasing temperature leads not only to a loss of coordinated water densities at the protein surface but also to a generally less structured hydration shell, as demonstrated by fewer water–protein and water–water hydrogen bonds, more dispersed protein–water and water–water correlations, and reduced enthalpic protein–water interactions. These findings provide the structural rationale for the decreasing contrast and Δ*R*_g_ values observed by our SAXS experiments and explicit-solvent SAXS calculations.

In summary, using a novel *T*-ramp SAXS setup at BioCARS, we investigated the GB3 domain and villin headpiece across a broad temperature range, spanning supercooled conditions to protein unfolding. When warming from cold temperatures, the SAXS data revealed a systematic decrease in the protein’s electron density contrasts and radii of gyration. MD simulations combined with explicit-solvent SAXS calculations showed excellent agreement with the experimental data, attributing these trends primarily to the temperature-dependent depletion of the hydration shell. The depletion is not solely caused by increased water disorder, as expected at increasing temperatures, but involves the partial displacement of surface-coordinated water molecules. Together, our SAXS experiments and simulations provide detailed structural insight into the protein hydration shell, highlighting its remarkable sensitivity to temperature and its potential influence on biological processes, such as cold denaturation, thermophoresis, and biomolecular phase separation.

## Methods

### *T*-ramp SAXS Experiments

The 35-residue villin headpiece subdomain (LSDED FKAVF GMTRS AFANL PLWKQ QHLKK EKGLF) was obtained from California Peptide Research Inc. The peptide was dialyzed against 20 mM acetate buffer (pH 4.9) with 150 mM NaCl. The GB3 domain (MQYKL VINGK TLKGE TTTKA VDAET AEKAF KQYAN DNGVD GVWTY DDATK TFTVTE) was expressed and purified as described previously^50^ and dissolved in 40 mM acetate buffer with 150 mM NaCl, 0.05% NaN_3_, and 5 mM DTT at pH 5.5.

Temperature-dependent small- and wide-angle X-ray scattering (SAXS-WAXS) data were acquired on the BioCARS 14IDB beamline at the Advanced Photon Source.^51–54^ Briefly, a peristaltic pump circulates in a closed-loop sample solution through a 560 mm long capillary (Polymicro TSP250350) that is supported on a home-built high-speed XYZ stage. To minimize scattering from the capillary walls, the region where X-rays pass through was HF-etched to a wall thickness of approximately 15–20 μm. X-rays passing through the capillary are scattered and detected by a large-area Rayonix MS340-HS detector positioned 186 mm downstream from the capillary. Thanks to the small, 0.51 mm diameter beamstop located near the midpoint between the sample and detector, the range of *q* accessible with 12 keV photons spans from 0.2 to the far-WAXS regime of 52 nm^–1^, albeit this study focused on the small-angle regime up to 3 nm^–1^ (Figure 1). The high-speed stage translates the sample capillary at a constant velocity over a ∼20 mm range, during which 40 X-ray shots are transmitted through the sample at ∼40 Hz with a separation of 0.5 mm along the capillary, thereby distributing the X-ray dosage over a large volume of the sample. During the return stroke, the X-ray scattering image is saved, and a fresh aliquot of solution is drawn from a ∼120 μL sample reservoir and pushed into the capillary. A homemade temperature controller ramped the sample temperature up and down between −16 and 120 °C at a rate of nearly 1 °C/s, repeating the ramp three times for each data set. Scattering data were acquired at each of three different concentrations (2.2, 6.9, 20 mg/mL for the villin headpiece and 2.7, 8.4, 24.7 mg/mL for GB3). To prevent boiling when ramping the capillary temperature to 120 °C, the sample reservoir was pressured with helium at 3 atm. Since we focused in this study on the hydration shell of folded proteins, we restricted further analysis to temperatures below 63 °C. They were averaged, extrapolated to the infinite dilution limit, and analyzed using the Guinier method to generate temperature-dependent *I*_0_ and *R*_g_ curves.

### Simulation Setup and Explicit-Solvent SAXS Calculations

Structures of the villin headpiece and the GB3 domain were taken from the protein data bank (PDB; codes 1yrf^55^ and 2oed,^42^ respectively). Crystal water was kept in the structures, and hydrogen atoms were added using pdb2gmx software.^56^ MD simulations were carried out using the GROMACS software, version 2021.7.^56^ Interactions of the proteins were described with the amber99SB-ildn force field (ff99SB-ildn).^57^ The starting structures were placed in a dodecahedral box, where the distance between the protein and box edges was at least 2.0 nm, and solvated in TIP4P/2005 ^41^ water. The charges of the proteins were neutralized by adding Na^+^ counterions. Additional salt was not added because (i) effects of NaCl on SAXS curves were small^39^ and (ii) force fields for ion–protein interactions were not well validated for wide temperature ranges and would, therefore, add uncertainty. After 400 steps of minimization with the steepest descent algorithm, the systems were equilibrated for 100 ps with harmonic position restraints applied to the heavy atoms of the proteins (force constant, 2000 kJ mol^–1^ nm^–2^). Subsequently, the production runs were carried out for 50 ns with harmonic position restraints applied to backbone atoms (force constant 2000 kJ mol^–1^ nm^–2^) at temperatures between 250 and 375 K in steps of 2.5 K. For each protein and temperature, four independent simulations were carried out. The equations of motion were integrated using the leapfrog algorithm.^58^ The temperature was controlled using velocity rescaling (τ = 1 ps).^59^ The pressure was controlled at 1 bar with the Berendsen barostat (τ = 1 ps)^60^ and the Parrinello–Rahman barostat (τ = 5 ps)^61^ during equilibration and production simulations, respectively. Since the experiments were carried out at 3 atm, we performed an additional series of simulations at the same pressure and found that the increased pressure had only a small effect on the results (Figure S9). The geometry of the water molecules was constrained with the SETTLE algorithm,^62^ and LINCS^63^ was used to constrain all other bond lengths. A time step of 2 fs was used. Dispersive interactions and short-range repulsion were described by a Lennard–Jones potential, which had a cutoff at 1 nm. The pressure and energy were corrected for missing dispersion corrections beyond the cutoff. Neighbor lists were updated with the Verlet scheme. Coulomb interactions were computed with the smooth particle-mesh Ewald method.^64,65^ We used a Fourier spacing of approximately 0.12 nm, which was optimized by the GROMACS mdrun module at the beginning of each simulation.

To compute the SAXS curves, 2500 simulation frames were used from the time interval between 0 and 50 ns. The SAXS calculations were performed using GROMACS-SWAXS, as also implemented by our web server WAXSiS.^25,45,66^ The implementation, documentation, and tutorials are available at https://gitlab.com/cbjh/gromacs-swaxs. A spatial envelope was built around the protein atoms in all frames. The distance between the protein and the envelope surface was at least 12 Å, such that all water atoms of the hydration shell were within the envelope. One envelope was generated for GB3, and one envelope was generated for villin; each was used throughout this study. Solvent atoms inside the envelope contributed to the calculated SAXS curves. Critically, the size of the envelope is not a fitting parameter, as it is not adjusted to match experimental data. Instead, the envelope is chosen to be large enough such that correlations between solvent densities inside and outside the envelope are due to bulk water.^25,67^ The buffer subtraction was carried out using 2498 simulation frames of a pure-water simulation box, which was simulated for 50 ns at the same temperature, as the protein simulation, and large enough to enclose the envelope. The orientational average was calculated by using 150 **q**-vectors for each absolute value of **q**. The solvent electron density was corrected to the temperature-dependent experimental value at the respective temperature such as 334 e/nm^3^ for 298.15 K, as described previously,^66^ using the empirical equation for water density as a function of temperature by Kell.^68^ In simulations at a pressure of 3 atm, the solvent density was, in addition to the equation by Kell, corrected by the isothermal compressibility of ambient water. No fitting parameters due to the hydration layer or excluded solvent were used, implying that the *R*_g_ values were not adjusted by fitting parameters. Nevertheless, the SAXS curves for GB3 and villin revealed reasonable agreement with the experimental curves, also over a wide *q* range (Figure S10). *I*_0_ and *R*_g_ values obtained via Guinier analysis from four independent replicas per temperature showed excellent agreement, as reflected by low standard errors relative to the corresponding mean values (*I*_0_: GB3 0.27%, villin 0.26%; *R*_g_: GB3 0.11%, villin 0.13%), as visualized by the good agreement from simulation results from individual simulations (Figures 2 and 3, colored dots).

Three-dimensional solvent densities around the GB3 domain and villin headpiece (Figures 1A and 4 and Movies S1–S4) were computed with the mdrun module of GROMACS-SWAXS using the environment variable GMX_WAXS_GRID_DENSITY and visualized with PyMOL (see Supporting Methods and Supporting ZIP folder).^69^ One-dimensional solvent densities around the protein (Figures S6A and S7A) were computed with the gmx genenv module of GROMACS-SWAXS. Here, three- and one-dimensional densities were computed from 12,500 frames extracted from a 25 ns simulation with restrained heavy atoms, which prevents the smearing of solvent densities near the protein surface due to side-chain fluctuations. The total number of excess electrons implied by the density profiles aligns with the literature (see Supporting Results). Protein–water interaction energies (Figures S6B and S7B) were computed as the sum of protein–water Lennard–Jones and short-range Coulomb interactions. Hydrogen bonds were computed with the gmx hbond module using standard settings.

RDFs between water oxygen atoms within the hydration shell were computed using an in-house modification of gmx genenv by using simulations with restraints on all backbone atoms. To this end, envelopes were constructed at distances of 0, 3, 5, or 7 Å from the van der Waals surface of the protein. For each MD frame of the production simulations, water oxygen atoms between (i) the envelope at 0 Å distance and (ii) the envelope at *x* Å distance were selected, where *x* ∈ {3, 5, 7}. Thereby, oxygen atoms within a distance of *x* Å from the protein were selected.

To decompose the total contrast into the contrast of the bare protein and the hydration shell, we defined the protein volume *V*_prot_ as the cavity volume calculated using the 3V volume calculator^70^ with a grid size of 0.16 Å and a probe radius of 1.4 Å, as used previously.^71,72^ Each volume was computed from 20 independent MD frames. The number of solute electrons *N*_e_^prot^ was taken from the atomic form factors at zero scattering angle, as defined by the Cromer–Mann parameters of the respective atoms.

## Supplementary Material













## Data Availability

A Gromacs variant GROMACS-SWAXS that implements explicit-solvent SAXS calculations is freely available at https://gitlab.com/cbjh/gromacs-swaxs. Documentation and tutorials are available at https://cbjh.gitlab.io/gromacs-swaxs-docs/.

## Notes

Absolute SAXS curves, Guinier analysis, simulation systems, analysis of solvent density correlations, hydrogen bonds, interaction energies, control simulations, and SAXS curve comparison to wider angles (Figures S1–S10) (PDF)
Three-dimensional electron density maps for all temperatures (Movie S1) (MP4)
Three-dimensional electron density maps for all temperatures (Movie S2) (MP4)
Three-dimensional electron density maps for all temperatures (Movie S3) (MP4)
Three-dimensional electron density maps for all temperatures (Movie S4) (MP4)
Electron densities and visualization scripts (ZIP)

## References

[1] Ball P. (2008). Water as an
Active Constituent in Cell Biology. Chem. Rev..

[2] Bellissent-Funel M.-C., Hassanali A., Havenith M., Henchman R., Pohl P., Sterpone F., van der Spoel D., Xu Y., Garcia A. E. (2016). Water Determines
the Structure and Dynamics of Proteins. Chem.
Rev..

[3] de
Grotthuss C. J. T. (1806). Sur La Décomposition de l’eau et Des
Corps Qu’elle Tient En Dissolution à l’aide de
l’électricité Galvanique. Ann. Chim..

[4] Mondal S., Mukherjee S., Bagchi B. (2017). Protein Hydration Dynamics: Much
Ado about Nothing?. J. Phys. Chem. Lett..

[5] Fogarty A. C., Duboué-Dijon E., Sterpone F., Hynes J. T., Laage D. (2013). Biomolecular
hydration dynamics: a jump model perspective. Chem. Soc. Rev..

[6] Bagchi B. (2005). Water Dynamics
in the Hydration Layer around Proteins and Micelles. Chem. Rev..

[7] Wüthrich K., Billeter M., Güntert P., Luginbühl P., Riek R., Wider G. (1996). NMR studies of the hydration of biological
macromolecules. Faraday Discuss..

[8] Crilly C. J., Eicher J. E., Warmuth O., Atkin J. M., Pielak G. J. (2021). Water’s
Variable Role in Protein Stability Uncovered by Liquid-Observed Vapor
Exchange NMR. Biochemistry.

[9] Laage D., Elsaesser T., Hynes J. T. (2017). Water Dynamics in the Hydration Shells
of Biomolecules. Chem. Rev..

[10] Ebbinghaus S., Kim S. J., Heyden M., Yu X., Heugen U., Gruebele M., Leitner D. M., Havenith M. (2007). An extended
dynamical
hydration shell around proteins. Proc. Natl.
Acad. Sci. U.S.A..

[11] Born B., Kim S. J., Ebbinghaus S., Gruebele M., Havenith M. (2009). The terahertz
dance of water with the proteins: the effect of protein flexibility
on the dynamical hydration shell of ubiquitin. Faraday Discuss..

[12] Sushko O., Dubrovka R., Donnan R. S. (2015). Sub-terahertz spectroscopy reveals
that proteins influence the properties of water at greater distances
than previously detected. J. Chem. Phys..

[13] Li T., Hassanali A. A., Kao Y.-T., Zhong D., Singer S. J. (2007). Hydration
Dynamics and Time Scales of Coupled Water-Protein Fluctuations. J. Am. Chem. Soc..

[14] Konstantinovsky D., Perets E. A., Santiago T., Velarde L., Hammes-Schiffer S., Yan E. C. Y. (2022). Detecting the First Hydration Shell Structure around
Biomolecules at Interfaces. ACS Cent. Sci..

[15] Furse K. E., Corcelli S. A. (2008). The Dynamics of
Water at DNA Interfaces: Computational
Studies of Hoechst 33258 Bound to DNA. J. Am.
Chem. Soc..

[16] Halle B., Nilsson L. (2009). Does the Dynamic Stokes
Shift Report on Slow Protein
Hydration Dynamics?. J. Phys. Chem. B.

[17] Yang M., Szyc Ł., Elsaesser T. (2011). Decelerated
Water Dynamics and Vibrational
Couplings of Hydrated DNA Mapped by Two-Dimensional Infrared Spectroscopy. J. Phys. Chem. B.

[18] Petersen C., Tielrooij K.-J., Bakker H. J. (2009). Strong temperature dependence of
water reorientation in hydrophobic hydration shells. J. Chem. Phys..

[19] Chatake T., Ostermann A., Kurihara K., Parak F. G., Niimura N. (2003). Hydration
in Proteins Observed by High-resolution Neutron Crystallography. Proteins: Struct., Funct., Bioinf..

[20] Nakasako M. (2004). Water-Protein
Interactions from High-Resolution Protein Crystallography. Philos. Trans. R. Soc., B.

[21] Svergun D. I., Richard S., Koch M. H. J., Sayers Z., Kuprin S., Zaccai G. (1998). Protein Hydration in
Solution: Experimental Observation
by x-Ray and Neutron Scattering. Proc. Natl.
Acad. Sci. U.S.A..

[22] Kim H. S., Martel A., Girard E., Moulin M., Härtlein M., Madern D., Blackledge M., Franzetti B., Gabel F. (2016). SAXS/SANS on Supercharged Proteins Reveals Residue-Specific Modifications
of the Hydration Shell. Biophys. J..

[23] Merzel F., Smith J. C. (2002). Is the First Hydration
Shell of Lysozyme of Higher
Density than Bulk Water?. Proc. Natl. Acad.
Sci. U.S.A..

[24] Köfinger J., Hummer G. (2013). Atomic-Resolution Structural Information from Scattering
Experiments on Macromolecules in Solution. Phys.
Rev. E.

[25] Chen P.-c., Hub J. S. (2014). Validating Solution
Ensembles from Molecular Dynamics
Simulation by Wide-Angle X-ray Scattering Data. Biophys. J..

[26] Persson F., Söderhjelm P., Halle B. (2018). The Geometry of Protein Hydration. J. Chem. Phys..

[27] Baldwin R. L. (1986). Temperature
Dependence of the Hydrophobic Interaction in Protein Folding. Proc. Natl. Acad. Sci. U.S.A..

[28] Huang D. M., Chandler D. (2000). Temperature and Length
Scale Dependence of Hydrophobic
Effects and Their Possible Implications for Protein Folding. Proc. Natl. Acad. Sci. U.S.A..

[29] Southall N. T., Dill K., Haymet A. D. J. (2002). A View
of the Hydrophobic Effect. J. Phys. Chem. B.

[30] Agashe V. R., Udgaonkar J. B. (1995). Thermodynamics
of Denaturation of Barstar: Evidence
for Cold Denaturation and Evaluation of the Interaction with Guanidine
Hydrochloride. Biochemistry.

[31] Dias C. L., Ala-Nissila T., Wong-Ekkabut J., Vattulainen I., Grant M., Karttunen M. (2010). The Hydrophobic
Effect and Its Role
in Cold Denaturation. Cryobiology.

[32] Wuttke R., Hofmann H., Nettels D., Borgia M. B., Mittal J., Best R. B., Schuler B. (2014). Temperature-Dependent
Solvation Modulates
the Dimensions of Disordered Proteins. Proc.
Natl. Acad. Sci. U.S.A..

[33] Dignon G. L., Zheng W., Kim Y. C., Mittal J. (2019). Temperature-Controlled
Liquid-Liquid Phase Separation of Disordered Proteins. ACS Cent. Sci..

[34] Ludwig C. (1856). Diffusion
Zwischen Ungleich Erwärmten Orten Gleich Zusammengesetzter
Lösungen. Sitzungber. Bayer. Akad. Wiss.,
Wien Math.-Naturwiss. Kl..

[35] Duhr S., Braun D. (2006). Why Molecules Move
along a Temperature Gradient. Proc. Natl. Acad.
Sci. U.S.A..

[36] Kumbhakar M., Goel T., Mukherjee T., Pal H. (2004). Role of Micellar Size
and Hydration on Solvation Dynamics: A Temperature Dependent Study
in Triton-X-100 and Brij-35 Micelles. J. Phys.
Chem. B.

[37] Ivanović M. T., Bruetzel L. K., Lipfert J., Hub J. S. (2018). Temperature-Dependent
Atomic Models of Detergent Micelles Refined against Small-Angle X-ray
Scattering Data. Angew. Chem., Int. Ed..

[38] Hub J. S. (2018). Interpreting
Solution X-ray Scattering Data Using Molecular Simulations. Curr. Opin. Struct. Biol..

[39] Linse J.-B., Hub J. S. (2023). Scrutinizing the Protein Hydration
Shell from Molecular
Dynamics Simulations against Consensus Small-Angle Scattering Data. Commun. Chem..

[40] Trewhella J., Vachette P., Bierma J. (2022). A Round-Robin Approach
Provides a Detailed Assessment of Biomolecular Small-Angle Scattering
Data Reproducibility and Yields Consensus Curves for Benchmarking. Acta Crystallogr., Sect. D: Struct. Biol..

[41] Abascal J. L. F., Vega C. (2005). A General Purpose Model
for the Condensed Phases of
Water: TIP4P/2005. J. Chem. Phys..

[42] Ulmer T. S., Ramirez B. E., Delaglio F., Bax A. (2003). Evaluation of Backbone
Proton Positions and Dynamics in a Small Protein by Liquid Crystal
NMR Spectroscopy. J. Am. Chem. Soc..

[43] Kubelka J., Eaton W. A., Hofrichter J. (2003). Experimental
Tests of Villin Subdomain
Folding Simulations. J. Mol. Biol..

[44] Lei H., Deng X., Wang Z., Duan Y. (2008). The Fast-Folding HP35
Double Mutant Has a Substantially Reduced Primary Folding Free Energy
Barrier. J. Chem. Phys..

[45] Knight C. J., Hub J. S. (2015). WAXSiS: AWeb Server for the Calculation of SAXS/WAXS
Curves Based on Explicit-Solvent Molecular Dynamics. Nucleic Acids Res..

[46] Brovchenko I., Krukau A., Smolin N., Oleinikova A., Geiger A., Winter R. (2005). Thermal breaking of spanning water
networks in the hydration shell of proteins. J. Chem. Phys..

[47] Smolin N., Winter R. (2008). Effect of Temperature,
Pressure, and Cosolvents on
Structural and Dynamic Properties of the Hydration Shell of SNase:
A Molecular Dynamics Computer Simulation Study. J. Phys. Chem. B.

[48] Tielrooij K.-J., Hunger J., Buchner R., Bonn M., Bakker H. J. (2010). Influence
of Concentration and Temperature on the Dynamics of Water in the Hydrophobic
Hydration Shell of Tetramethylurea. J. Am. Chem.
Soc..

[49] Ben-Amotz D. (2019). Hydration-Shell
Vibrational Spectroscopy. J. Am. Chem. Soc..

[50] Tian P., Louis J. M., Baber J. L., Aniana A., Best R. B. (2018). Co-Evolutionary
Fitness Landscapes for Sequence Design. Angew.
Chem. Int. Ed..

[51] Graber T., Anderson S., Brewer H. (2011). BioCARS: A Synchrotron
Resource for Time-Resolved X-ray Science. J.
Synchrotron Radiat..

[52] Cho H. S., Schotte F., Stadnytskyi V., DiChiara A., Henning R., Anfinrud P. (2018). Dynamics of Quaternary
Structure Transitions in R-State
Carbonmonoxyhemoglobin Unveiled in Time-Resolved X-ray Scattering
Patterns Following a Temperature Jump. J. Phys.
Chem. B.

[53] Cho H. S., Schotte F., Stadnytskyi V., Anfinrud P. (2021). Time-Resolved X-ray
Scattering Studies of Proteins. Curr. Opin.
Struct. Biol..

[54] Henning R. W., Kosheleva I., Šrajer V., Kim I.-S., Zoellner E., Ranganathan R. (2024). BioCARS: Synchrotron Facility for Probing Structural
Dynamics of Biological Macromolecules. Struct.
Dyn..

[55] Chiu T. K., Kubelka J., Herbst-Irmer R., Eaton W. A., Hofrichter J., Davies D. R. (2005). High-resolution x-ray crystal structures of the villin
headpiece subdomain, an ultrafast folding protein. Proc. Natl. Acad. Sci. U.S.A..

[56] Abraham M. J., Murtola T., Schulz R., Páll S., Smith J. C., Hess B., Lindahl E. (2015). GROMACS: High
performance
molecular simulations through multi-level parallelism from laptops
to supercomputers. SoftwareX.

[57] Lindorff-Larsen K., Piana S., Palmo K., Maragakis P., Klepeis J. L., Dror R. O., Shaw D. E. (2010). Improved
side-chain
torsion potentials for the Amber ff99SB protein force field. Proteins.

[58] Hockney R., Goel S., Eastwood J. (1974). Quiet high-resolution computer models
of a plasma. J. Comput. Phys..

[59] Bussi G., Donadio D., Parrinello M. (2007). Canonical
sampling through velocity
rescaling. J. Chem. Phys..

[60] Berendsen H. J. C., Postma J. P. M., van Gunsteren W. F., DiNola A., Haak J. R. (1984). Molecular
dynamics with coupling to an external bath. J. Chem. Phys..

[61] Parrinello M., Rahman A. (1981). Polymorphic transitions in single crystals: A new molecular
dynamics method. J. Appl. Phys..

[62] Miyamoto S., Kollman P. A. (1992). Settle: An analytical
version of the SHAKE and RATTLE
algorithm for rigid water models. J. Comput.
Chem..

[63] Hess B. (2008). P-LINCS: A
Parallel Linear Constraint Solver for Molecular Simulation. J. Chem. Theory Comput..

[64] Darden T., York D., Pedersen L. (1993). Particle mesh Ewald: An N·log­(N)
method for Ewald sums in large systems. J. Chem.
Phys..

[65] Essmann U., Perera L., Berkowitz M. L., Darden T., Lee H., Pedersen L. G. (1995). A smooth particle
mesh Ewald method. J. Chem. Phys..

[66] Chen P.-c., Shevchuk R., Strnad F. M., Lorenz C., Karge L., Gilles R., Stadler A. M., Hennig J., Hub J. S. (2019). Combined
Small-Angle X-ray and Neutron Scattering Restraints in Molecular Dynamics
Simulations. J. Chem. Theory Comput..

[67] Park S., Bardhan J. P., Roux B., Makowski L. (2009). Simulated X-Ray Scattering
of Protein Solutions Using Explicit-Solvent Models. J. Chem. Phys..

[68] Kell G. S. (1975). Density,
Thermal Expansivity, and Compressibility of Liquid Water from 0. Deg.
to 150. Deg.. Correlations and Tables for Atmospheric Pressure and
Saturation Reviewed and Expressed on 1968 Temperature Scale. J. Chem. Eng. Data.

[69] Schrödinger, LLC . The PyMOL Molecular Graphics System.

[70] Voss N. R., Gerstein M. (2010). 3V: cavity, channel and cleft volume calculator and
extractor. Nucleic Acids Res..

[71] Shumilina, A. In A Fast Method for Determination of Solvent-Exposed Atoms and Its Possible Applications for Implicit Solvent Models, Computational Science and Its Applications – ICCSA 2005; Springer Berlin Heidelberg: Berlin, Heidelberg, 2005; pp 1075–1082.

[72] Lee B., Richards F. (1971). The interpretation
of protein structures: Estimation
of static accessibility. J. Mol. Biol..

