# Supplementary material for: Depletion of the Protein Hydration Shell with Increasing Temperature Observed by Small-Angle X‑ray Scattering and Molecular Simulations
Source: J Am Chem Soc. 2025 Dec 11;147(51):47117–25. doi: 10.1021/jacs.5c13497 (PMC12750538; doi:10.1021/jacs.5c13497)
Supplement: Supplementary file 1 [file ja5c13497_si_001.pdf]

Supplementary material for: Depletion of the protein hydration shell with increasing temperature observed by small-angle X-ray scattering and molecular simulations

Johanna-Barbara Linse,[†] Hyun Sun Cho,[‡] Friedrich Schotte,[‡] Philip A. Anfinrud,[*,‡,¶] and Jochen S. Hub[*,†,¶]

†*Theoretical Physics and Center for Biophysics, Saarland University, Saarbrücken, 66123, Germany*

‡*Laboratory of Chemical Physics, National Institute of Diabetes and Digestive and Kidney Diseases, National Institutes of Health, Bethesda, MD 20892, USA*

¶*Corresponding author*

E-mail: philipa@intra.niddk.nih.gov; jochen.hub@uni-saarland.de

Supporting Results

Solvent density profiles as function of distance from the protein surface

To illustrate the solvent structure within the hydration layer, we computed the solvent density as a function of the distance from the van der Waals surface of the protein, taken from simulations with restrained heavy atoms (Fig. S6A, S7A). Here, the restraints on heavy atoms were used to exclude spatial fluctuations of protein side chains, which would lead to a smearing of the water density and, thereby, to a smearing of the density peaks in Figs. S6A and S7A. The density profiles were computed with the GROMACS-SWAXS module gmx genenv with option -od (Fig. S6A, S7A). To this end, gmx genenv constructs a series of spatial envelopes around the protein surface at distances up to 12Å in steps of 0.1Å and counts the number of solvent electrons within the 0.1Å thick concentric volume elements, as averaged over 12,500 simulation frames taken from a 25 ns simulation for each temperature.

Critically, since such removal of side chain fluctuations likely influences the packing of water on the protein surface, the density profiles obtained from such simulations with restrained heavy atoms are not suitable for obtaining the overall excess number of water molecules for comparison with our SAXS experiments. Nevertheless, to test whether the density profiles align qualitatively with excess densities reported in the literature, we computed the excess number of electrons implied by the profiles via

$$\Delta N_e = \sum_i (\rho(R_i) - \rho_{\mathrm{bulk}})\Delta V_i,$$

where R_i is the binned distance from the van der Waals surface of the protein, $\rho(R_i)$ the density, ρ_{bulk} the bulk solvent density defined as the average of $\rho(R_i)$ in the $R_i > 9$ Å region, and ΔV_i denotes volume elements between concentric pairs of envelopes constructed around the protein by gmx genenv, as also used to compute the densities. Taking the profile for GB3 at 300 K as an example (Fig. S6A, orange curve), this leads to $\Delta N_e = 401 \pm 4$ excess electrons in the hydration shell, or, taking the approximate surface area of the GB3 domain

of 3800 Å^2 obtained with the gmx sasa module,[1] an excess of $0.105 \pm 0.1\,e$ per Å^2 of protein surface.

Assuming that these excess electrons would be uniformly distributed in a layer of a thickness of 3 Å, the excess of $0.105\,e$/Å^2 would correspond to uniform excess density of $0.035\,e$/Å^3 or ∼10% of the bulk density of $0.334\,e$/Å^3. This value aligns with common assumptions made by implicit-solvent SAXS predictors that model the hydration shell as a uniform 3Å layer with an excess density of 10–15% of the bulk density.[2]

Supporting Methods

Solvent density calculations

Three-dimensional (3D) solvent densities were computed with the rerun functionality of the GROMACS-SWAXS mdrun module (gmx mdrun -rerun), as activated with the environment variable GMX_WAXS_GRID_DENSITY. See https://cbjh.gitlab.io/gromacs-swaxs-docs/ for documentation. In brief, mdrun places a cubic grid with a 1Å spacing across the volume of the envelope. Then, electron densities are assigned to the grid based on the number of electrons of atoms, here taken from the Cromer-Mann parameters. Densities are assigned based on nuclei position. Densities are averaged over MD frames. With the environment variable GMX_WAXS_GRID_DENSITY_MODE=2, only solvent densities (but not solute densities) are computed, as used here. The mdrun module writes the resulting density to a CUBE file to allow visualization with PyMOL.[70]

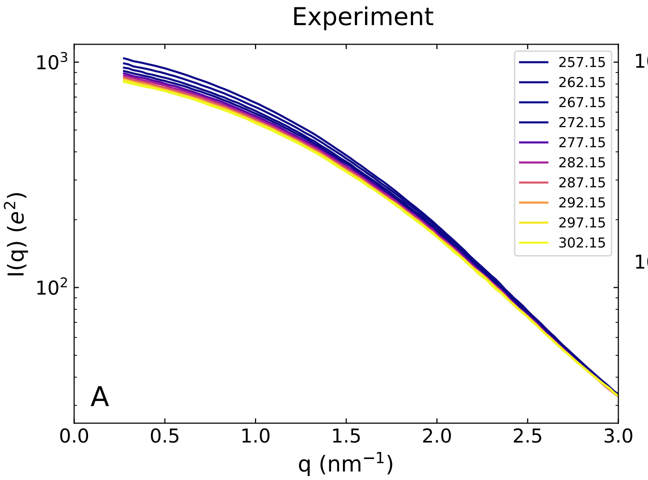

Figure S1: SAXS curves of the GB3 domain from (A) experiment, extrapolated to infinite dilution, and (B) MD simulation over the temperature range shown in the figure legend.

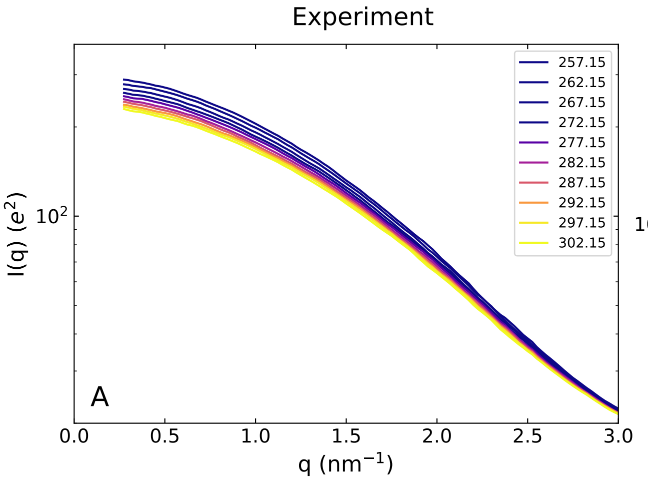

Figure S2: SAXS curves of villin head headpiece from (A) experiment, extrapolated to infinite dilution, and (B) MD simulation over the temperature range shown in the figure legend.

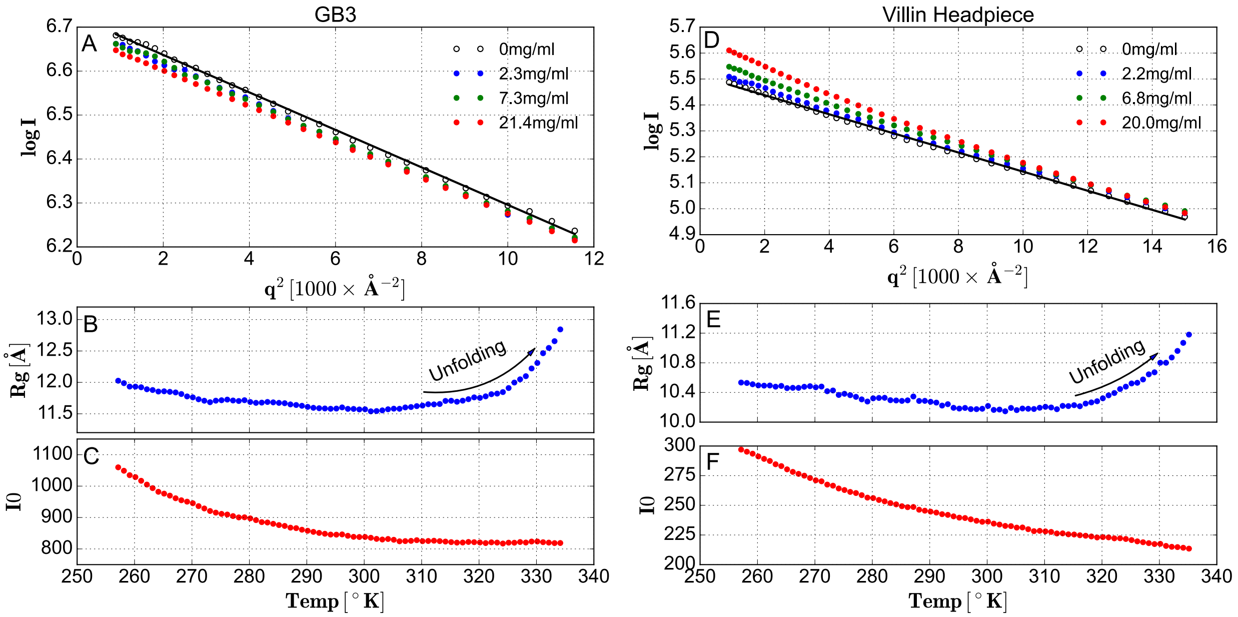

Figure S3: Guinier analysis of GB3 and Villin SAXS scattering. Guinier analysis of GB3 and Villin SAXS scattering. (A) Concentration-dependent SAXS scattering of GB3 at 295 K. The concentration-dependent, buffer-subtracted scattering amplitudes I_i were divided by their relative concentration rc_i to put them on the same scale, i.e., rc_i is approximately [1 (red), 1/3 (green), 1/9 (blue)]. Differences between the normalized scattering curves arise from the concentration-dependent packing structure factor, whose effects can be mitigated by extrapolating the experimental curves to the limit of infinite dilution (open circles). This extrapolated curve corresponds to the infinite dilution limit of a statistically-weighted linear fit of (I_i/rc_i) as a function of concentration at each value of q. The solid line is a Guinier fit of the infinite dilution limit. (B) Radius of gyration R_g from Guinier fits of the infinite dilution limit calculated from concentration-dependent scattering data acquired in 1 deg steps from 257–335 K. (C) I_0 from corresponding Guinier fits. (D–F) Same as (A–C) but for villin headpiece. Due to attractive interparticle interactions, the scattering intensities of Villin headpiece exhibit opposite concentration behavior compared to GB3. The decrease of R_g and I_0 with increasing temperature (while folded) in both GB3 and Villin headpiece correlates with depletion of waters in their hydration shell.

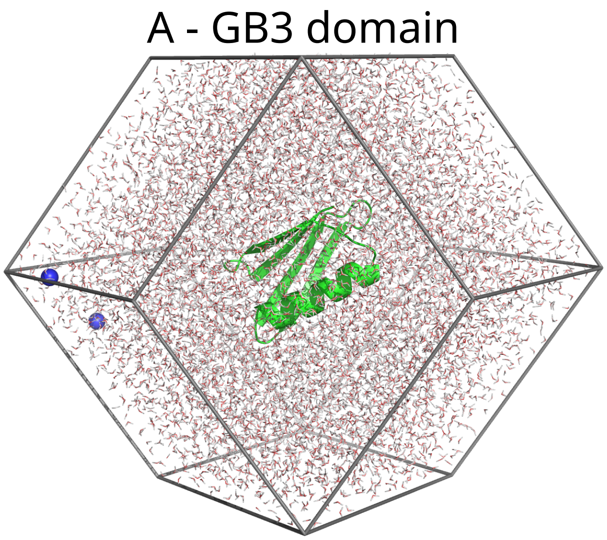

Figure S4: Simulation systems of (A) the GB3 domain and (B) villin headpiece in dodecaheral simulation boxes. Proteins are shown in green cartoon representation, water as red/white sticks, and counter ions as blue spheres.

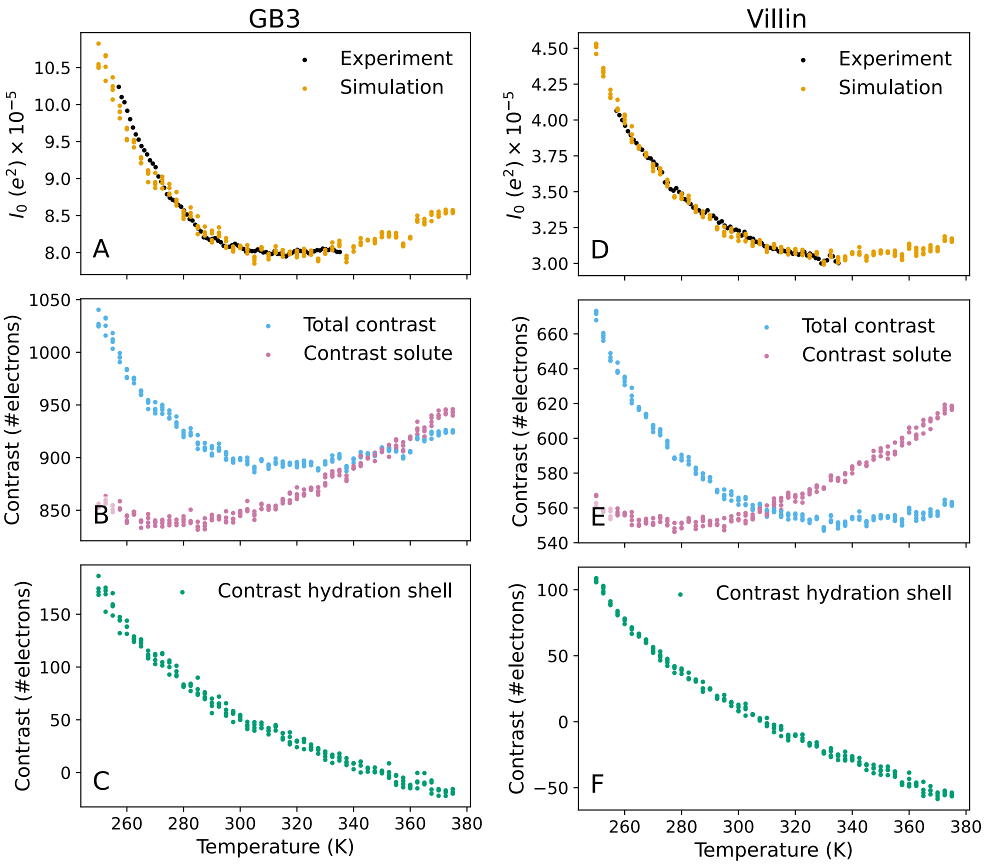

Figure S5: Forward scattering I_0 and analysis of contrast in number of electrons versus temperature for (A–C) the GB3 domain and (D–F) villin headpiece. Same data as presented in Fig. 2, however presented to directly illustrate our analysis. (A/D) I_0 from experiment (black) and backbone-restrained MD simulations (orange) versus temperature. The experimental data was scaled by a constant factor to the simulation data in the temperature range below 303 K. (B/E) Total contrast in number of electrons computed as $I_0^{1/2}$ from MD simulation (blue) and contrast $\Delta N_\mathrm{e}^\mathrm{prot}$ due to the solute (pink). (C/F) Contrast of the hydration shell $\Delta N_\mathrm{e}^\mathrm{hs}$ given as the difference between the total contrast (B/E, blue) and solute contrast (B/E, pink). Colored dots in panels (A–F) indicate simulation results from four independent simulation replicates per temperature.

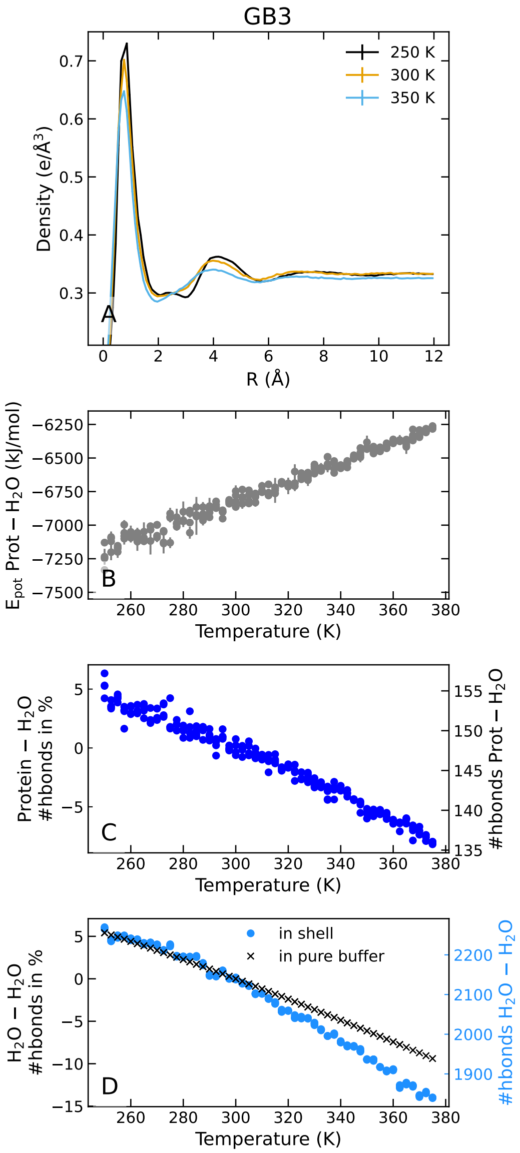

Figure S6: Analysis of the hydration shell of the GB3 domain. (A) Solvent density versus distance from the Van-der-Waals surface of the protein as averaged over the protein surface at three temperatures (see legend), computed from simulations with restrained heavy atoms. (B) Protein–water interaction energy versus temperature computed as sum of Lennard-Jones and short-range Coulomb energies. (C) Number of protein–water hydrogen bonds, plotted either as total number of hydrogen bonds (right ordinate) or as change of number of hydrogen bonds relative to 300 K (left ordinate). (D) Number of water–water hydrogen bonds in the hydration shell (blue dots), defined as water within a distance of 9 Å from the protein surface. Plotted as number of hydrogen bonds (right ordinate) and relative to 300 K (left ordinate). For reference, the relative change of the number of water–water hydrogen bonds is shown for bulk water (black crosses, left black ordinate). Dots in panels B/C indicate simulation results from four independent simulation replicates per temperature.

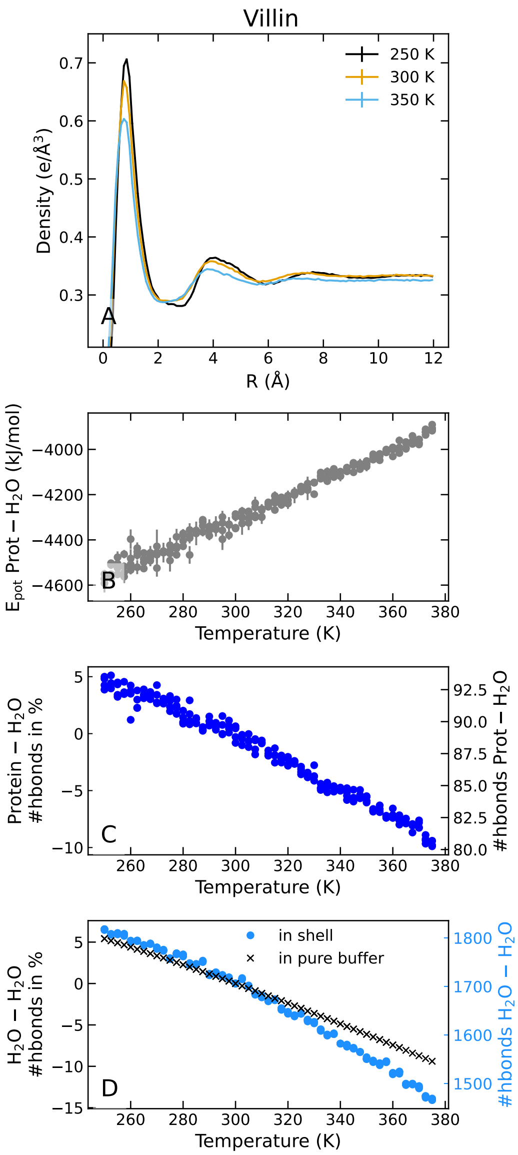

Figure S7: Analysis of the hydration shell of villin. (A) Solvent density versus distance from the Van-der-Waals surface of the protein as averaged over the protein surface at three temperatures (see legend), computed from simulations with restrained heavy atoms. (B) Protein–water interaction energy versus temperature computed as sum of Lennard-Jones and short-range Coulomb energies. (C) Number of protein–water hydrogen bonds, plotted either as total number of hydrogen bonds (right ordinate) or as change of number of hydrogen bonds relative to 300 K (left ordinate). (D) Number of water–water hydrogen bonds in the hydration shell (blue dots), defined as water within a distance of 9 Å from the protein surface. Plotted as number of hydrogen bonds (right ordinate) and relative to 300 K (left ordinate). For reference, the relative change of the number of water–water hydrogen bonds is shown for bulk water (black crosses, left black ordinate). Dots in panels B/C indicate simulation results from four independent simulation replicates per temperature.

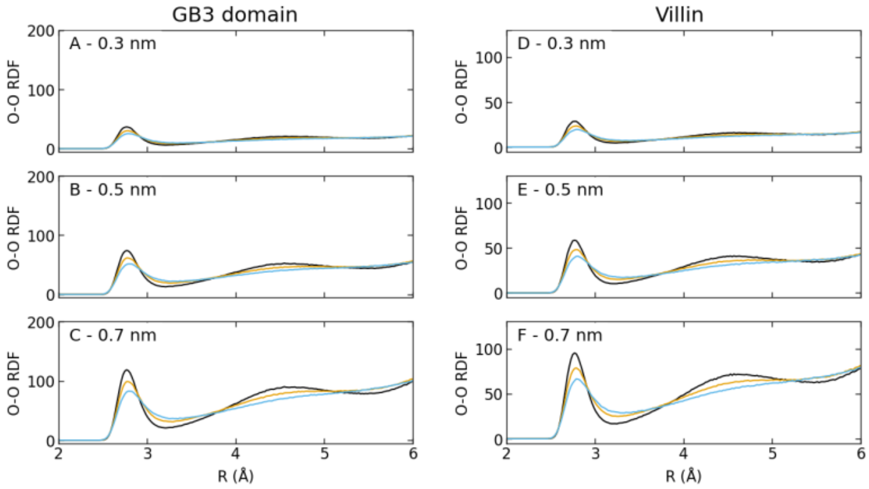

Figure S8: Analysis of the internal water structure within the hydration shell. (A) Non-normalized radial distribution functions (RDFs) between pairs of water oxygen atoms (O–O) for (A–C) the GB3 domain or (D–F) villin headpiece. RDFs are shown for temperatures of 250 K (black), 300 K (orange), and 350 K (blue). RDFs were computed for O–O pairs within distances of (A/D) 0.3 nm, (B/E) 0.5 nm, or (C/F) 0.7 nm from the protein surface. Peaks of the RDFs decay with increasing temperature, indicating a loss of water structure within the hydration shell.

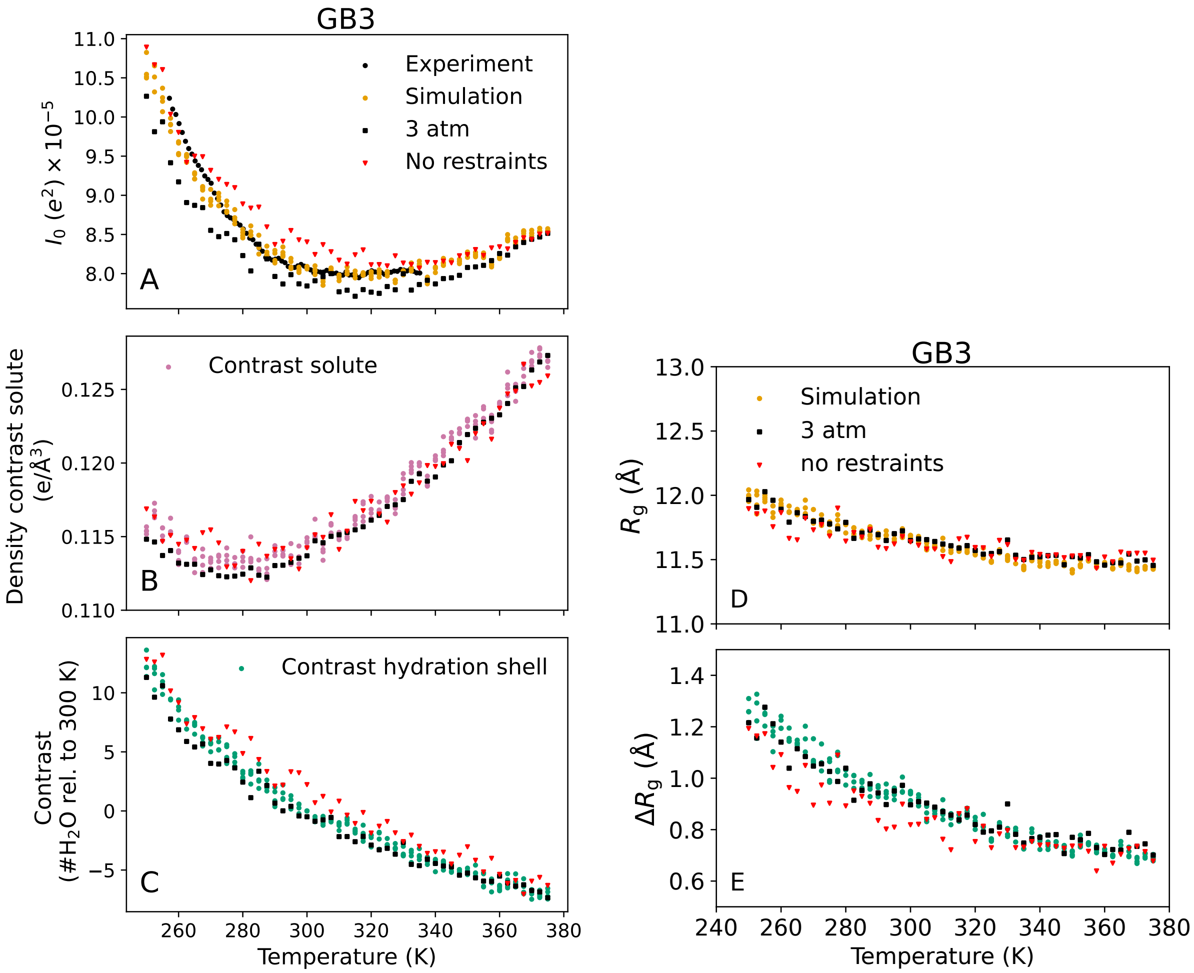

Figure S9: Control simulations with the GB3 domain of 25 ns each for testing the effects of simulating at an increased pressure of 3 atm (black squares) or simulating without positions restraints (red triangles down) instead of simulating with ambient pressure and with position restraints on backbone atoms (yellow, purple, or green circles). (A) Forward scattering I_0 from MD simulations versus temperature, (B) density contrast of the bare protein, and (C) contrast of the hydration shell, according to the analysis in Fig. 2A–C. (D) Radius of gyration R_g from MD simulations and (E) difference ΔR_g between R_g from Guinier analysis (including hydration shell contributions) and R_g of the bare protein, according to the analysis in Fig. 3A/B. Simulating at 3 atm instead of 1 atm or simulating without position restraints has only a small effect on the results. Notably, in unrestrained simulations, the GB3 domain did not unfold at temperatures within simulation time of 25 ns, rationalizing the good agreement with the restrained simulations across the entire temperature range.

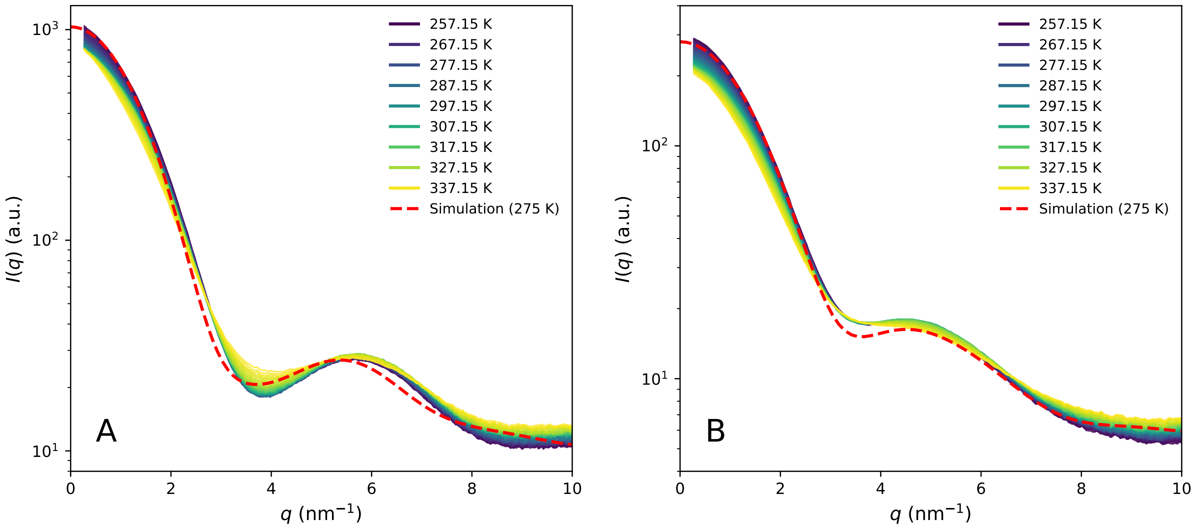

Figure S10: Comparison of SAXS curves from experiment (color gradient) with an example curve from simulations at 275 K (red dashed) for (A) the GB3 domain and (B) villin headpiece to wider angles up to $q = 10\,\mathrm{nm}^{-1}$. The legend shows the color for a few representative temperatures. Reasonable agreement is found, despite the fact that the hydration shell and, thereby, the radius of gyration is not fitted against the data. Instead, for this figure only, the calculated curve was fitted to the experimental curve at the closest temperature via $I_{\mathrm{fitted}}(q) = f I_{\mathrm{exp}}(q) + c$ by adjusting the absolute scale f and a constant offset c. While the absolute scale was likewise adjusted for comparison of I_0 in Figs. 2A/D and S9A, a constant offset was not adjusted for other analyses of this study.

References

(1) Abraham, M. J.; Murtola, T.; Schulz, R.; Páll, S.; Smith, J. C.; Hess, B.; Lindahl, E. GROMACS: High performance molecular simulations through multi-level parallelism from laptops to supercomputers. *SoftwareX* **2015**, *1-2*, 19 – 25.

(2) Svergun, D.; Barberato, C.; Koch, M. H. J. CRYSOL – a Program to Evaluate X-ray Solution Scattering of Biological Macromolecules from Atomic Coordinates. *J. Appl. Cryst.* **1995**, *28*, 768–773.
